# Systems science for universal health coverage

**DOI:** 10.2471/BLT.17.192542

**Published:** 2017-07-01

**Authors:** Timothy G Evans, Marie Paule Kieny

**Affiliations:** a Health, Nutrition and Population, The World Bank, Washington, United States of America.; b World Health Organization, avenue Appia 20, 1211 Geneva 27, Switzerland.

It is 20 years since an international consultation in Lejondal, Sweden, highlighted the need for more and better research “to understand and improve how societies organize themselves in achieving collective health goals, and how different actors interact in the policy and implementation processes to contribute to policy outcomes.”^1^ One outcome was the creation of the Alliance for Health Policy and Systems Research.^1^

There have since been several important milestones on the path towards more and better health systems research. The Alliance for Health Policy and Systems Research found its home at the World Health Organization (WHO) in 1998 and subsequently issued a series of reports on health systems.^2^^,^^3^ Health systems research entered the mainstream of global health policy – not only at WHO^4^ and the World Bank,^5^ but also with the founding of Gavi, the Vaccine Alliance, in 2000,^6^ and the Global Fund to Fight AIDS, Tuberculosis and Malaria in 2002.^6^ Ministerial meetings on health research, in 2004 and 2008,^7^ increased the demand for – and the national priority given to – such research. In 2010, a biennial global symposium on health systems research was initiated and this development led to the first global society of health systems researchers: Health Systems Global. More recent efforts in low- and middle-income countries – e.g. the establishment of a knowledge platform in India^8^ – are indicative of a shift towards systems thinking at national level.

Such encouraging developments need to be carefully balanced against areas where progress has not met expectations. The development of national capacity for financing and institutional leadership of health policy and systems research has been slow.^9^ Such research also remains constrained by several common challenges – e.g. the complexity of health systems, the context specificity of research findings and the large numbers of disciplines and epistemological perspectives involved.^10^

Looking forward, however, we see a brighter future for health policy and systems research. Sustainable development goal (SDG) 3 – particularly its target of universal health coverage – has promoted the establishment of common performance metrics against which the relative effectiveness of alternative policies and programmes can be compared. There is a growing global interdependence in health – as reflected by infectious diseases such as the Ebola virus – that requires systems’ knowledge and public health investments in global readiness. The rapid growth seen in the health sector is raising systems-wide demand for knowledge and innovation to improve value for money and overcome inefficiencies related to high prices, lack of equity and poor quality. Finally, the paradigm shift towards patients being recognized as the co-designers and co-creators of their own health and health care is raising the demand for evidence that would make it possible to navigate the promises and perils of accountable care, personalized medicine and big data.

How can health policy and systems research seize this favourable context and contribute more effectively to universal health coverage, greater health security, improved value in health and effective engagement of citizens?

First, we need a comprehensive review of the progress that has been made and the progress that might be anticipated in the future.

Second, we need to accelerate the development of capacity and opportunity for health policy and systems research in low- and middle-income countries. A focus on results should be extended to cultivate a culture of learning for improvement.^11^

Third, we need a revitalized approach to stewardship that reflects the pluralistic reality of contemporary health systems and prioritizes health policy and systems research across all countries. Through such stewardship, we need to nurture a shared agenda around: (i) cross-country comparative research on health systems issues of common concern; (ii) agreements on the prioritization of global public goods related to the development of measures and methods for health policy and systems research; and (iii) best practices in evidence synthesis and knowledge translation.

We need to appreciate the progress made over the last two decades and develop strategies to use systems science in progressing towards universal health coverage.
